# THREE-YEAR EVALUATION OF THE DETECTION OF ELDER ABUSE THROUGH EMERGENCY CARE TECHNICIANS PROJECT

**DOI:** 10.1093/geroni/igz038.639

**Published:** 2019-11-08

**Authors:** Michael B Cannell, Doug Livingston, Megin Parayil

**Affiliations:** 1 University of Texas Health Science Center at Houston School of Public Health, Dallas, Texas, United States; 2 Emory University School of Public Health, Atlanta, Georgia, United States

## Abstract

EA is difficult to detect and often goes unreported. To address this important public health issue we developed the DETECT screening tool, which assists paramedics and EMT’s (medics) with determining whether or not to report a case of potential EA to APS while in the field providing medical services. In the current study, we investigate the effects of the DETECT screening tool on changes in reports of EA to APS over a period of approximately 3 years (January 2015 to March 2018). We used a differences in differences in differences (DDD) design to estimate the effect of the DETECT tool on both the number of reports for EA reports made each week as well as the probability of a report being validated by APS. After adjusting for changes in the number of EA reports non-medics and medics outside the service area, there were on average three times as many reports among medics after the implementation of the DETECT screening tool (RR=3.03, 95% CI:[2.06, 4.46]). No differences were seen in the probability of a valid report attributable to DETECT.

